# Functional Specialization and Collaboration of cDC2 Subsets in CD4+ T Cell Priming and Differentiation

**DOI:** 10.1111/imr.70069

**Published:** 2025-11-07

**Authors:** Naoya Tatsumi, Ariel Tjitropranoto, Alejandro Davila‐Pagan, Yosuke Kumamoto

**Affiliations:** ^1^ Center for Immunity and Inflammation Rutgers New Jersey Medical School Newark New Jersey USA; ^2^ Department of Pathology, Immunology and Laboratory Medicine Rutgers New Jersey Medical School Newark New Jersey USA

**Keywords:** cell differentiation, cell lineages and subsets, dendritic cells, processes, Th1/Th2/Th17 cells

## Abstract

Dendritic cells (DCs) play a crucial role in bridging innate and adaptive immunity by presenting antigens to prime antigen‐specific T cells. Conventional DC (cDC) type 2 cells (cDC2s) are a phenotypically heterogeneous population of DCs highly capable of presenting antigens to CD4+ T cells. Studies found functional differences between different cDC2 subsets in regulating effector CD4+ T helper (Th) cells, but collaboration between different cDC subsets has also been suggested. In this review, we discuss recent advances in understanding the subset‐specific role of cDC2s in regulating Th cells.

## 1 Introduction

Differentiation of antigen‐specific CD4+ T cells into effector T helper (Th) cells is fundamental to shaping the nature of the subsequent adaptive immune response. Upon priming, multiple T cell‐intrinsic and extrinsic factors affect their differentiation propensity to different types of Th cells such as Th type 1 (Th1), Th2, Th17, T follicular helper (Tfh), and T regulatory (Treg) cells. Conventional dendritic cells (cDCs) are the primary professional antigen‐presenting cells (APCs) that convey activation and differentiation signals to naïve T cells. Although all cDCs share a common developmental origin that is different from monocytes and other myeloid progenitors, mature cDCs consist of phenotypically and developmentally distinct subsets that preferentially induce different types of Th cells.

In both mice and humans, cDCs are generally classified into two main subsets, cDC1 and cDC2 cells, based on the requirement of the transcription factors BATF3 and IRF8 for the development of the former but not the latter [1, 2]. While cDC1 cells are relatively homogeneous, cDC2 cells show substantial heterogeneity in their phenotype, which is further escalated by the fact that monocytes and their precursors can also acquire a cDC2‐like phenotype known as DC3 or monocyte‐derived DCs (mo‐DCs) [3, 4]. Recent studies have highlighted functional differences in CD4+ T cell priming among the subsets of cDC2 and related cells. Here, we review the recent advances in understanding their subset‐specific roles and mechanisms in Th cell differentiation.

## 2 cDC Subsets

### 2.1 Differentiation of cDC Subsets

Following the discovery of cDCs more than five decades ago [5], earlier studies on cDCs described their phenotypic heterogeneity and its correlation with their function, such as the association of differences in cytokine production and localization with their preferential priming of CD8+ and CD4+ T cells and their effector subsets [6, 7, 8, 9, 10, 11, 12, 13]. A seminal work by Dudziak et al. demonstrated that the subset‐specific preference in CD8+ vs. CD4+ T cell priming is dictated by the collective expression of antigen‐processing and presentation molecules rather than by subset‐specific antigen‐uptake receptors that are often used as subset‐defining markers [14], leading to a notion that cDCs can be generally classified into functionally dichotomous subsets [15]. The functional dichotomy of cDC subsets was further corroborated by the identification of BATF3 as a transcription factor required for the development of cDC populations critical for cross‐presenting antigens to CD8+ T cells across multiple organs in mice [16, 17]. Subsequently it was proposed to classify all cDC subsets that require BATF3 for their development as cDC1 and the remainder of cDCs as cDC2 regardless of differences in cell surface marker phenotype [1]. Phenotypically, cDC1 cells generally express a chemokine receptor XCR1 and a transcription factor IRF8, while cDC2 cells express CD172a (SIRPα) and IRF4 as mutually exclusive markers in mice among other subset‐specific molecules [18].

During myelopoiesis, cDCs arise from pre‐cDCs, a cDC‐committed precursor population that originates in the bone marrow (BM) from monocyte‐dendritic cell progenitors (MDPs) [19]. Before exiting the BM, MDPs differentiate either into the specified monocyte progenitor (common monocyte progenitors, cMoPs) or into common dendritic cell progenitors (CDPs) that further differentiate into committed precursors for cDC1 (pre‐cDC1), cDC2 (pre‐cDC2), or plasmacytoid DCs (pDCs) [19, 20, 21]. BATF3 does not directly drive pre‐cDC1 differentiation but instead supports the maintenance of their cDC1 identity by sustaining the expression of IRF8, another transcription factor required for the lineage specification of pre‐cDC1 cells [22]. These subset‐committed pre‐cDCs exit the BM, seed peripheral non‐lymphoid tissues, and proliferate and differentiate locally into mature cDC1s and cDC2s [21, 23]. Once in peripheral organs, cDCs constantly take up exogenous and endogenous (self) antigens in the tissue and migrate to the draining lymph node (dLN) through the lymphatics, where they present antigens to T cells and are often called “migratory” cDCs. Alternatively, pre‐cDCs migrate directly into the secondary lymphoid organs such as the LNs and spleen via the blood and form a lymphoid tissue‐resident DC population [24], which then acquires blood‐ and lymph‐borne antigens and presents them to T cells. In the steady‐state LNs, migratory DCs can be distinguished from the LN‐resident DCs based on their more mature phenotype with higher expression of MHC class II (MHCII).

In addition to cDCs derived from the pre‐cDCs, it has long been known that monocytes can acquire a cDC‐like phenotype with high expression of MHCII and CD11c and low expression of Ly6C upon exposure to GM‐CSF in vitro or under inflammatory conditions in vivo [25, 26, 27, 28]. With more precise lineage tracing, adoptive transfer and single‐cell analysis techniques, recent studies identified cDC‐like cell subsets known as mo‐DC (also known as inflammatory DCs under certain conditions) and DC3s in vivo, which are derived from monocytes and from pro‐DC3 cells, an MDP‐derived intermediate precursor population distinct from pre‐cDC or cMoP, respectively, in both mice and humans [29, 30, 31, 32, 33, 34, 35]. While their subset‐specific functions remain elusive, these studies generally agree upon phenotypic similarity between cDC2s, mo‐DCs and DC3s, with the exception of a few cell surface markers specifically expressed in one or the other. Furthermore, recent studies have identified yet another subset of cells that arise from lymphoid progenitors through a pDC‐like precursor population but exhibit a mature cell phenotype similar to pre‐cDC2‐derived cDC2s [32, 36, 37, 38, 39, 40]. Thus, unlike cDC1 cells that arise from the developmentally homogeneous pre‐cDC1 population, cells of multiple lineages can acquire a cDC2‐like phenotype, making it often difficult, and sometimes even biologically irrelevant [41], to estimate the developmental origin of cDC2‐like cells solely based on the expression of a few cell surface markers. Therefore, for the purpose of this review, “cDC2” refers to all cDC2‐like cells unless their developmental origin is specifically mentioned.

### 2.2 Heterogeneity and Developmental Requirements of cDC2s

Aside from the heterogeneity among cDC2‐like cells associated with their developmental origin, heterogeneity within the pre‐cDC2‐derived cDC2s has also been described (Table 1). The transcription factor IRF4 is generally expressed by cDC2s but not by cDC1s and plays various roles in the development and function of different cDC2 subsets [18]. For instance, IRF4 is required for the development of splenic CD11b hi CD4+ CD8α− and lung CD11b+ CD172a+ CD24+ cDC2s [42, 43, 44, 45, 46], whereas it is dispensable for the development but crucial for the migration of dermal CD11b+ cDC2s in the skin [47, 48]. As such, conditional deletion of *Irf4* in CD11c+ cells in mice results in a significant reduction of various cDC2 subsets including CD11b+ DCs in the lung, mediastinal LNs and skin‐dLN as well as CD103+ CD11b+ cDC2s in the small intestinal lamina propria and mesenteric LN, whereas intestinal CD103− CD11b+ cDC2s are less affected [18, 43, 46, 48, 49, 50, 51]. Thus, while IRF4 is often considered the signature transcription factor for cDC2s [2], subset‐dependent heterogeneity exists in its function. In contrast, Liu et al. recently reported near complete loss of cDC2s and a reciprocal increase of cDC1s in the spleen, skin‐dLN and mesenteric LNs in mice lacking specific enhancer loci in the *Zeb2* transcription factor, which provide binding sites for other transcription factors NFIL3, C/EBPα and C/EBPβ (*Zeb2* ^∆1+2+3^ mice), suggesting the critical requirement of ZEB2 in cDC2 fate commitment [52]. However, it remains unclear if ZEB2 also plays a role in the cDC2‐like cells originated from monocytic precursors, as these mice are also devoid of monocytes [52].

**TABLE 1:** Developmental requirement of cDC subsets and associated Th responses.

Regulator	Mouse genetic model	Effect on cDC subset	Organs tested	Including CD301b+ cDC2s?^a^	Impact on Th responses	Antigen/pathogen	Route	Ref.
BATF3	*Batf3* ^*−/−*^	CD8α+ cDC1 (missing) XCR1+ cDC1 (missing) CD103+ cDC1 (missing)	Spleen, skin‐dLN	No	CTL: ↓ proliferation, IFNγ in Ag‐specific CD8+ T	WNV	s.c.	17
					CTL: ↓ tumor rejection	Sarcoma cells	s.c.	17
			Skin	No	Th1: ↓ IFNγ+ Ag‐specific CD4+ T	*C. albicans*	t.p.	80
			Skin	No	Th1: ↓ IFNγ+ CD4+ T	*L. major*	i.d.	81
ZEB2	*Zeb2* enhancer mutant (*Zeb2* ^Δ1+2+3^)	CD172a+ XCR1*−* cDC2 (missing)	Skin‐dLN	Yes	NA	NA	NA	52
			MesentericLN	Yes	Th2: ↓ GATA3+, IL‐4+, IL‐5+, IL‐13+ CD4+ T	*H. polygyrus*	o.g.	52
			Spleen	No	Th2: ↓ GATA3^+^ CD4+ T	*H. polygyrus*	o.g.	52
IRF4	*Itgax* (CD11c)‐Cre; *Irf4* ^*fl/fl*^	CD11b+ CD172a+ CD24+ cDC2 (missing)	Lung	Yes	Th2: ↓ IL‐4, IL‐5, IL‐13 in lung‐dLN, ↓ inflammation to HDM	HDM	i.n.	46, 55
					Th17: ↓ IL‐17A+ CD4+ T	Steady‐state	NA	43
					Th17: ↓ IL‐17A+ CD4+ T	*A. fumigatus*	i.n.	43
					Tfh: ↓ CXCR5+ PD‐1+ Ag‐specific CD4+ T	OVA + LPS	i.n.	50
					pTreg: ↓ FOXP3+ CD4+ T	Steady‐state	NA	93
		CD11b+ CD103+ cDC2 (missing)	Small intestine	No	Th2: ↓ IL‐4+, IL‐5+, IL‐13+ CD4+ T	*N. brasiliensis*	s.c.	48
					Th2: ↓ IL‐4+, IL‐5+, IL‐13+ CD4+ T	*S. mansoni*	i.c.	153
					Th17: ↓ IL‐17A+ CD4+ T	Steady‐state	NA	43
					Th17: ↓ Protection against *C. rodentium*	*C. rodentium*	o.i.	54, 55
					Th17: ↓ IL‐17A+ Ag‐specific CD4+ T	OVA + anti‐CD40 + LPS	i.p.	51
		CD11b hi CD4+ CD8α− cDC2 (missing)	Spleen	No	Th17: ↓ IL‐17A+ CD4+ T	*A. fumigatus*	i.n.	43
		CD11b+ cDC2 (migration defect)	Skin	Yes	Th2: ↓ Protection against *S. mansoni*	*S. mansoni*	s.c.	55
					Th2: ↓ IL‐4+ Ag‐specific CD4+ T	OVA + papain	s.c.	48
NOTCH2	*Itgax*‐Cre; *Notch2* ^*fl/fl*^	CD103+ CD11b+ cDC2 (missing)	Small intestine	No	Th17: ↓ IL‐17A+ CD4+ T upon PMA/ionomycin stimulation	Steady‐state	NA	53
					Th17: ↓ Protection against *C. rodentium*	*C. rodentium*	o.i.	54, 55
		ESAM hi cDC2 (missing)	Spleen	No	Tfh: ↓ CXCR5+ PD‐1+ CD4+ T	Sheep erythrocytes	i.v.	156
KLF4	*Itgax*‐Cre; *Klf4* ^*fl/fl*^	CD172a+ CD24+ cDC2 (missing)	Lung	Yes	Th2: ↓ HDM‐induced inflammation	HDM	i.n.	55
		CD11b+ CD24+ cDC2 (missing)	Skin	NA	Th2: ↓ protection against *S. mansoni*	*S. mansoni*	s.c.	55
		CD11b*−* CD24− migratory cDC2 (missing)	skin LN	No	Th2: ↓ protection against *S. mansoni*	*S. mansoni*	s.c.	55
		CD172a+ cDC2 (partial loss)	Intestine	Yes	NA (Normal protecation against *C. rodentium*)	*C. rodentium*	o.i.	55
BCL6	*Itgax*‐Cre; *Bcl6* ^*fl/fl*^	CD103+ CD11b+ cDC2 (missing) XCR1+ cDC1 (ectopic CD11b expression)	Intestine	No	Th17: ↓ IL‐17A+ CD4+ T	*C. rodentium*	o.i.	157
	*Clec9a* ^+*/Cre*^; *Bcl6* ^*fl/fl*^	ESAM hi cDC2 (missing)	Spleen	No	Tfh: ↓ CXCR5+ PD‐1+ Ag‐specific CD4+ T	OVA + alum	i.p.	157
					Tfr: ↓ FOXP3+ CXCR5+ PD‐1+ CD4+ T	KLH + alum	i.p.	157

Abbreviations: HDM, house dust mite; i.c., intracolonic; i.d., intradermal; i.n., intranasal; i.p., intraperitoneal; i.v., intravenous; KLH, Keyhole limpet haemocyanin; NA, not addressed; o.g., oral gavage; o.i., oral inoculation; s.c., subcutaneous; t.p., topical application; WMV, West Nile virus.

^a^ This column indicates whether the affected cDC subset includes CD301b+ cDC2s in WT mice.

Besides the variable requirement of IRF4, the NOTCH2 receptor and the transcription factor KLF4 are required for the development of some cDC2s in a subset‐specific manner. For instance, conditional deletion of *Notch2* in CD11c+ cells in mice leads to a selective loss of splenic ESAM hi cDC2s and CD11b+ CD103+ cDC2s in the intestinal lamina propria and mesenteric LNs, while CD11b+ CD103− cDC2s remain intact [53, 54]. In contrast, conditional deletion of *Klf4* in CD11c+ cells selectively depletes CD24+ CD301b+ cDC2s in the lung, CD11b+ CD24+ in the dermis, and migratory CD11b− CD24− cDC2s in the skin‐dLNs [55]. Brown et al. recently described two distinct cDC2 subsets, cDC2A and cDC2B, in both mice and humans based on the selective expression of a transcription factor T‐bet in the cDC2A population, which also overlap phenotypically with the ESAM hi and ESAM lo splenic cDC2s, respectively [56]. The distinction between these cDC2 subsets traces back to pre‐cDC2s, whose differentiation into mature cDC2A and cDC2B cells requires NOTCH2 and KLF4, respectively [39, 57]. Thus, NOTCH2 and KLF4 appear to play a general role in bifurcating the cDC2 population into two distinct subsets [2], though their role in the diversification of cDC2‐like cells outside of the pre‐cDC2 lineage remains less clear.

While the developmental requirement of NOTCH2 and KLF4 may dictate overall bifurcation of pre‐cDC2‐derived cDC2s into two main groups, further phenotypic heterogeneity exists among cDC2s, which is often tissue‐specific and sometimes without definitive correlation with their developmental origin. For instance, the NOTCH2‐dependent cDC2A cells include ESAM hi cDC2s and CD11b+ CD103+ cDC2s in the spleen and intestinal lamina propria, respectively [53, 54]. However, the ESAM hi cDC2s are found only in the spleen and phenotypically distinct from any other cDC2 subsets in other organs, suggesting spleen‐specific adaptation rather than a universal cDC2 feature shared across other tissues [29]. In contrast, a C‐type lectin CLEC12A (MICL) appears to be universally expressed at high levels in NOTCH2‐independent cDC2B cells [29, 39, 57], whereas another C‐type lectin CD301b (*Mgl2*) is expressed by a specific subpopulation, but not all, of cDC2B cells across all organs [29, 56, 57, 58, 59, 60]. However, some studies have shown the expression of CD301b in a subset of pre‐cDC2‐derived cDC2s, DC3s, or mo‐DCs in particular organs and/or under certain inflammatory conditions [29, 57, 61, 62, 63], indicating that its expression may not be restricted to cells of a specific developmental origin. Nevertheless, CD301b+ cDC2s form a major subset among migratory cDC2s in peripheral organs while they are relatively rare among the splenic and LN‐resident cDC2s [29, 58, 59, 60, 64], suggesting that their differentiation may be boosted by the peripheral tissue microenvironment. Thus, these studies collectively indicate that, in addition to the developmental heterogeneity, cDC2 subsets acquire phenotypic heterogeneity likely through environmental cues.

## 3 Regulation of Effector CD4+ Th Cell Differentiation

### 3.1 Dynamics of Th Cell Priming and Differentiation In Vivo

CD4+ T cell activation and differentiation into effector cells begin with cDC‐mediated priming in the secondary lymphoid organs such as the dLNs. Given the rarity of antigen‐specific clones, the immune system must efficiently scan polyclonal T cells to mount rapid responses. Naïve lymphocytes continuously recirculate between blood and LNs patrolling for cognate antigens. Meanwhile, antigen‐bearing cDCs migrate from peripheral tissues via afferent lymphatics and localize to appropriate niches in the dLNs. The initial CD4+ T cell activation occurs in confined areas of the paracortex near the high endothelial venules (HEVs) [13, 65, 66, 67]. Upon cognate peptide–MHC II recognition, naïve CD4+ T cells rapidly proliferate and differentiate into distinct effector Th cell subsets. Most of these effector cells leave the LNs to reach infected tissues, whereas Tfh precursors upregulate the chemokine receptor CXCR5, which guides them to the boundary areas between the T cell zone and B cell follicles (T‐B border) and subsequently into the B cell follicles [67].

Multiple types of APCs cooperate to complete CD4+ T cell priming. Itano et al. showed that soluble antigens injected into the skin are presented in the dLNs in two waves, an early wave within 4 h by mixed migratory APCs including LC, cDC1s, and cDC2s that capture the passively diffused antigen in the dLN, and a later wave at around 24 h by dermis‐derived migratory cDC2s acquiring antigen at the immunization site [68]. Surgical removal of the injection site prevents the second wave but does not impair CD4+ T cell proliferation, indicating that initial priming can occur without antigen acquisition at the tissue site. However, priming efficiency upon subcutaneous immunization with a soluble protein antigen is severely reduced in CCR7‐deficient mice, in which the tissue‐to‐dLN migration of APCs is impaired, as well as in CD11c‐Aβb mice, in which the MHCII expression is restricted to LN‐resident DCs, underscoring the essential role of skin‐derived APC migration [69, 70]. Lethally irradiated wild‐type (WT) mice reconstituted with CD11c‐Aβb BM cells, but not those reconstituted with CCR7‐deficient BM cells, exhibit normal CD4+ T cell priming. This suggests that radio‐resistant migratory APCs, most likely LCs, cooperate with LN‐resident cDCs to overcome the priming defect in the CD11c‐Aβb mice. However, MHC‐II expression by LCs alone is insufficient to support CD4+ T cell priming in this model [69]. These findings indicate that distinct waves of APCs cooperate to orchestrate effective CD4+ T cell priming.

### 3.2 Differentiation of Th Cell Subsets

Following TCR activation, antigen‐specific naïve CD4+ T cells differentiate into distinct Th subsets such as Th1, Th2, Th17, Tfh, and peripherally derived Treg (pTreg) cells, each of which is defined by a specific transcription program that triggers signature cytokines to coordinate immune responses tailored to eliminate particular types of pathogens [71, 72]. The master transcriptional regulators T‐bet, GATA3, RORγt, BCL6 and FOXP3 specify the Th1, Th2, Th17, Tfh and pTreg effector fate, which is crucial for their interferon (IFN)γ production, type 2 cytokine production, IL‐17A production, CXCR5 and IL‐21 upregulation for helping B cells, and execution of immunoregulatory functions, respectively. Thus, the regulation of Th cell differentiation early after the antigen exposure shapes the subsequent adaptive immune response.

Differentiation of naive CD4+ T cells into effector Th subsets is driven by multiple factors, including signals both dependent (antigen dose, TCR signal strength, co‐stimulation) and independent (cytokines, cell adhesion molecules) of active engagement with the cognate antigen [72]. While critical cytokines involved in this process have been extensively studied in vitro, their induction in vivo depends also on the dynamics of unique signals provided primarily by cDCs [71, 73]. The differentiation into Th1 cells is driven by IL‐12, which directly induces T‐bet expression via STAT4 independently of the IFNγ‐STAT1 signaling, but the latter reinforces T‐bet expression and stabilizes the Th1 phenotype [74, 75]. cDC1s generally produce a higher amount of IL‐12 [76, 77, 78, 79, 80, 81], but other DC subsets can also serve as a critical source of IL‐12 to prime Th1 responses [82, 83, 84].

The differentiation of Th2 cells is partly a self‐amplifying process, as the Th2 signature cytokine IL‐4 drives their own differentiation by inducing GATA3 through activating STAT6 [85]. As such, IL‐4 is required for inducing Th2 cell differentiation in vitro [86, 87]. In contrast, studies in vivo have found only a moderate, if any, requirement of the IL‐4‐STAT6 signaling for Th2 differentiation in mice infected with a helminth parasite *Nippostrongylus brasiliensis* or those exposed to house dust mite [88, 89]. However, Th2 cells are markedly reduced in IL‐4‐deficient mice in the peripheral organs despite their modest reduction in the dLN [88], suggesting that IL‐4 plays a more significant role in their maintenance in non‐lymphoid tissues than in the Th2 cell fate commitment per se. Notably, cDCs are generally required for priming Th2 cells [90], but they are not considered an efficient source of IL‐4 [73, 91], though some studies found IL‐4 expression in specific cDC subsets [92, 93]. Accordingly, cDC‐specific IL‐4 production is dispensable for Th2 polarization [94, 95]. Thus, cDC‐derived cytokine that directly induces the Th2 cell fate in naïve CD4+ T cells is yet to be clearly identified [73].

IL‐17‐producing Th17 cells can be induced in vitro by IL‐6 and TGF‐β, which is further augmented by adding TNF‐α and IL‐1β [96, 97, 98], or by IL‐6 and IL‐23 in the presence of IL‐1β without TGF‐β [99]. cDCs serve as a critical source of these cytokines to drive Th17 differentiation in vivo [100, 101, 102]. However, compared with Th1 and Th2 cells [103], the Th17 cells generated in vitro remain ‘flexible’ even after multiple rounds of stimulation and can be switched to Th1 and Th2 when transferred into respective culture conditions [104], implying a role of environmental cues for their maintenance.

In addition to its role in Th17 cell differentiation, TGF‐β also plays a crucial role in the function and development of Tregs by promoting the induction of FOXP3 [105]. Naïve conventional CD4+ T cells can be converted to FOXP3+ Tregs in vitro by TCR activation in the presence of TGF‐β1 and IL‐2 [106, 107]. Several studies have demonstrated the requirement of cDCs in maintaining peripheral tissue homeostasis by inducing antigen‐specific pTregs [108, 109, 110], as cDCs produce a multitude of immunoregulatory molecules such as IL‐10, TGF‐β, retinoic acid and PD‐1 ligands in a subset‐ and context‐dependent manner [110, 111, 112, 113].

Unlike other effector Th cell subsets that execute their function in peripheral organs, Tfh cells primarily reside in the B cell follicles in the secondary lymphoid organs to help B cells differentiate into germinal center B (GCB) cells and produce high‐affinity antibodies. As such, the differentiation of Tfh cells is dictated by localized differentiation cues from cDCs and B cells that are critically dependent on their positioning in the secondary lymphoid organs [114]. The transcription repressor BCL6 is specifically required and sufficient for the development of Tfh cells and suppresses alternative Th fates [115, 116, 117, 118]. Consistent with the universal production of high‐affinity antibodies regardless of the type of pathogen, the bifurcation between Tfh and non‐Tfh effector cells is a common framework across all types of immune responses [119, 120]. This bifurcation is partly a CD4+ T cell‐autonomous process intrinsic to the TCR signal intensity [121, 122] and is accelerated by IL‐2, which potently suppresses BCL6 and thereby bifurcates the Tfh precursors that produce but do not respond to IL‐2 from the non‐Tfh effector cells that do not produce IL‐2 but respond to it [123, 124, 125, 126]. Nevertheless, recent studies showed that cDCs either promote or suppress the differentiation of Tfh cells in a subset‐specific manner [127].

Upon TCR engagement, IL‐2 and its receptor subunit CD25 (IL‐2Rα) are rapidly upregulated by the activated T cells themselves. While IL‐2 enhances the expression of CD25, its downstream signal through STAT5 suppresses the expression of IL‐2, which enables cell‐intrinsic control of IL‐2 production [128, 129, 130, 131]. Moreover, the local concentration of IL‐2 in the T cell priming environment is tightly regulated by the surrounding Tregs, which naturally express high levels of CD25 and are heavily dependent on this cytokine for their development and survival [132, 133]. In addition to the suppression of Tfh and promotion of Treg fate, the IL‐2‐STAT5 signaling supports Th1 differentiation by directly promoting the expression of Th1‐associated genes such as *Il12rb*, *Tbx21* (T‐bet) and *Ifng*, though IL‐2 alone does not skew the CD4+ T cells toward Th1 effectors [134, 135]. Likewise, the IL‐2–STAT5 axis directly upregulates GATA3 and IL‐4Rα in Th cells, establishing the IL‐4–IL‐4Rα–STAT6–GATA3 positive feedback loop essential for reinforcing the Th2 identity [136, 137]. In contrast, the IL‐2‐induced STAT5 suppresses Th17 differentiation by competing with STAT3 [138, 139], a transcription factor required for Th17 differentiation at the downstream of IL‐6, IL‐21 and IL‐23 [139, 140, 141, 142, 143, 144]. Notably, while activated T cells themselves are generally considered as the major source of IL‐2, cDCs can also produce IL‐2 upon stimulation [145, 146, 147, 148], and the role of cDC‐derived IL‐2 in regulating Th cells has been highlighted in recent studies [149, 150, 151].

Together, these studies have established the T cell‐intrinsic signaling network and T cell‐extrinsic environmental cues as critical determinants in shaping effector Th cell fate decisions, enabling context‐specific immune responses or tolerance.

### 3.3 Regulation of Th Cell Differentiation by cDC Subsets

Consistent with the specific cytokine requirements for Th subsets and the subset‐specific cytokine expression patterns in cDC subsets, mice developmentally lacking specific cDC subsets demonstrated subset‐specific roles of cDCs in CD4+ T cell responses (Table 1). Most notably, the cDC1‐null *Batf3* ^*−/−*^ mice have been widely used to address the role of cDC1s. In addition to the poor CD8+ T cell response observed in a wide variety of models due to the lack of cDC1‐dependent antigen cross‐presentation [152], Th1 differentiation upon infection with *Candida albicans* , *Leishmania major* or *Toxoplasma gondii* is impaired in those mice, likely reflecting their high IL‐12 production capacity [79, 80, 81], whereas Th2 differentiation upon infection with helminth parasites *Schistosoma mansoni* or *Heligmosomoides polygyrus* is enhanced [78].

The *Zeb2* ^∆1+2+3^ mice, which lack all CD172a+ XCR1*−* cDC2s and monocytes in the skin‐dLNs, mesenteric LNs and spleen, exhibit diminished Th2 cell differentiation upon infection with *H. polygyrus* [52]. Likewise, mice with CD11c+ cell‐specific deletion of *Irf4* or *Klf4*, which have developmental and/or functional defects in a subset of pan‐cDC2s and cDC2Bs, respectively, also show impaired Th2 cell differentiation in multiple organs following exposure to allergens or helminth parasites [46, 48, 55, 153, 154]. Notably, the CD11c+ cell‐specific *Klf4*‐deficient mice mount normal cytotoxic T cell, Th1 and Th17 responses against herpes simplex virus, *T. gondii*, or *Citrobacter rodentium* infections [55]. By contrast, the *Irf4* deficiency in CD11c+ cells results in impaired Th17 differentiation in the intestine and mesenteric LNs at the steady state or following intraperitoneal immunization with ovalbumin (OVA) with anti‐CD40 and LPS, as well as in the lung upon intranasal infection with *Aspergillus fumigatus* [43, 51]. Furthermore, these mice also show impaired Tfh differentiation when immunized subcutaneously or intranasally with OVA plus LPS [50], and sporadic CD4+ T cell expansion due to a loss of the pTreg compartment [93]. These diverse defects demonstrate the functional heterogeneity of IRF4‐dependent cDC2 cells [2].

The defective Th17 and Tfh differentiation in the CD11c+ cell‐specific *Irf4*‐deficient mice is also phenocopied in mice with CD11c+ cell‐specific *Notch2* deficiency, which lack cDC2A cells including small intestinal CD103+ CD11b+ cDC2s, splenic ESAM hi cDC2s, and a minor population of LN‐resident ESAM+ cDC2s [53, 54]. These mice show reduced numbers of Th17 cells in the small intestinal lamina propria at the steady state and are more susceptible to oral infection with *C. rodentium* [53, 54], while they develop antigen‐specific Th17 cells normally upon intranasal infection with *Streptococcus pyogenes* [155], suggesting tissue‐dependent function of NOTCH2‐dependent cDC2s. In addition, intravenous immunization of these mice with sheep red blood cells results in markedly reduced Tfh cell differentiation, which is not observed in mice with CD11c+ cell‐specific *Klf4* deletion [156]. The impaired Th17 and Tfh responses are likewise observed in mice lacking BCL6 specifically in cDCs, which also show a reduction in intestinal CD103+ CD11b+ cDC2s and splenic ESAM hi cDC2s, though these mice have an additional cDC population with an unusual XCR1+ CD11b+ cDC1‐cDC2 hybrid phenotype [157].

These findings indicate a division of labor among cDC subsets in Th cell differentiation. In particular, cDC2s comprise functionally heterogeneous populations, where subset‐specific requirements for NOTCH2 and KLF4 define their tissue‐specific roles in promoting Th17 and Th2 responses, respectively, in an organ‐dependent manner.

## 4 CD301b+ cDC2s as a Functionally Specialized cDC2 Subset in Th Cell Differentiation

### 4.1 Phenotype and Development of CD301b+ cDC2s

The C‐type lectin CD301b, encoded by the *Mgl2* gene in mice, has been identified originally as a marker selectively expressed in migratory CD11b+ CD207 (Langerin)− dermal cDC2s in the skin and skin‐dLNs [58]. CD301b+ cells in the skin are found exclusively in the dermis and are excluded from the epithelial layer inhabited by Langerhans cells (LCs). Similarly, CD301b+ cells in mucosal barrier organs are also localized specifically to the submucosa beneath the epithelial layer [60]. They are present in virtually all peripheral organs but are rare, compared to those in peripheral tissues, in the spleen [29, 59, 64]. In the skin‐dLNs and elsewhere, CD301b expression is restricted to CD11b+ cDC2s, with no overlap with markers for cDC1, LC and mo‐DC such as XCR1, CD103, CD207, CD326 and Ly6C [29, 58, 59, 150]. Consistently, the *Mgl2* expression in the skin‐dLNs, examined by the inducible tdTomato reporter in the Mgl2+/Cre; Rosa26lsl‐tdTomato mice, is restricted to a subset of PD‐L2+ migratory (MHCII hi) cDC2s, with minimal leakage into the PD‐L2− migratory DC2s, LCs, XCR1+ migratory cDC1s, and LN‐resident cDCs. In addition, CD301b+ and CD301b− cDC2s in the LNs of naïve mice express CD86 and MHCII at a comparable level, suggesting that the expression of CD301b is not a simple indicator for maturation status but is rather marking a distinct subset of CD11b+ cDC2s, as also shown in other organs [29, 150]. These CD11b+ CD301b+ cDC2s express high levels of PD‐L2 and IRF4, the latter of which is required for their migration to the skin‐dLN but appears to be dispensable for their development in the skin per se [48]. In contrast, mice lacking IRF4 in CD11c+ cells show a significant reduction of CD301b+ cDC2s in both lung and lung‐dLN upon viral infection despite the normal number of the total lung cDC2s [49], suggesting a context‐dependent requirement of IRF4 for their development. Likewise, KLF4 deficiency in CD11c+ cells in mice results in a significant reduction of CD301b+ cDC2s in the lung and liver, while it causes selective loss of CD11b− cDC2s, which do not express CD301b, in the skin‐dLN, also suggesting an organ‐specific requirement of KLF4 for their differentiation [55]. Like other cDCs, the development of dermal CD301b+ cDC2s is severely impaired in mice lacking FLT3, a cytokine receptor critical for cDC differentiation, but is also partially abrogated in mice treated with a neutralizing antibody against GM‐CSF [61]. Together, the expression of CD301b in mice marks a major and specific subset of migratory cDC2s that express high levels of CD11b and PD‐L2 and require IRF4, KLF4, FLT3 and GM‐CSF for their differentiation in a context‐dependent manner.

In mice, two homologous genes *Clec10a* (also known as *Mgl1*) and *Mgl2*, located adjacent to each other on Chromosome 11, separately encode CD301a and CD301b proteins, respectively. In contrast, humans have a single CD301 (MGL/CLEC10A) protein encoded by the *CLEC10A* gene, which has slightly higher homology with the murine *Mgl2* than with the *Clec10a* gene [158]. The mouse CD301a/MGL1 protein is expressed in broader cell subsets than the CD301b protein, including medullary sinus macrophages, CD301b+ cDC2s, as well as B220+ pDC‐like cells [58, 64, 159], which presumably correspond to the recently identified pDC‐like cDC2s or their precursors [32, 36, 37, 38, 39, 40]. While the data in human peripheral tissues are still scarce, studies on human blood and lymphoid organs have thus far indicated selective and universal expression of the human CD301 (CLEC10A) protein by CD1c+ DCs, the human equivalent of murine cDC2B cells [56, 160, 161], though others have argued their similarity to murine pre‐cDC2 and pro‐DC3s [30]. Given that murine CD301b protein is restricted to a specific subpopulation of cDC2B cells [29, 56, 57], the human CD301 protein appears to be expressed in broader cDC2 subsets than the mouse CD301b protein.

### 4.2 Subset‐Specific Depletion of CD301b+ DCs

The mice lacking specific cDC subsets due to global or myeloid‐specific gene deletion have undoubtedly provided important insights into the developmental mechanism of cDC subsets and their function in vivo (Table 1). However, one caveat of such an approach is that it is sometimes unclear whether the observed phenotype is due to the loss of the target cDC subset or to the loss of the molecule in the remaining cells. For example, the cDC1‐null *Batf3* ^*−/−*^ mice have been widely used to examine the requirement of cDC1s in numerous models, but recent studies found a critical role of T cell‐intrinsic BATF3 in CD8+ T cell memory formation [162, 163], potentially complicating the interpretation of previous studies. Thus, it is plausible to have complementary approaches whenever possible to definitively demonstrate the role of a cDC subset. A powerful alternative approach is to directly deplete a mature cDC subset without complicating the transcriptional regulation of their development. Since murine cells are naturally resistant to diphtheria toxin (DT) due to a lack of a functional receptor, mouse models artificially expressing the DT receptor (DTR) in a specific cell type are often used to selectively ablate the target cells in vivo by administrating DT. Alternatively, targeted expression of DT in a specific cell type results in autonomous depletion of the target cells. These models allow investigation into the role of a specific cell type in vivo even when their developmental mechanism is unknown or not defined by a single transcriptional factor. Since first utilized by Jung et al. in the CD11c‐DTR transgenic mouse to deplete pan‐DCs [164], several mouse strains have been generated to selectively deplete a specific DC population based on the DT/DTR system using various genetic drivers [165, 166], including the *Mgl2*‐DTR mice, in which CD301b+ cDC2s are selectively depleted upon treatment with DT [60]. Given the developmental and phenotypic heterogeneity among cDC2s, this approach serves as a valuable complement to the models with conditional deletion of *Irf4*, *Klf4*, or *Notch2* to study the role of cDC2 subsets.

### 4.3 CD301b+ cDC2s in Th2 Cell Differentiation

CD301b+ cDC2s were originally characterized as a specific cDC subset in the murine dermis that plays a crucial role in fluorescein isothiocyanate (FITC)‐induced contact hypersensitivity, a Th2‐biased model of T cell‐dependent contact dermatitis [58, 167]. LCs (CD103− CD207+ CD301b−), CD103+ cDC1s (CD103+ CD207+ CD301b−) and CD301b+ cDC2s (CD103− CD207− CD301b+) constitute the majority of migratory APCs in mouse skin. LCs are localized exclusively in the epidermis while the other two subsets are found only in the dermis in the skin, whereas in the LNs CD301b+ cDC2s are localized to the T‐B border near the HEVs while LCs and CD103+ DCs are localized deep in the T cell zone [58, 168, 169]. Adoptively transferring CD301b+ cDC2s, but not LCs or dermal cDC1s, isolated from the skin‐dLNs of FITC‐sensitized mice is sufficient for inducing dermatitis upon secondary challenge with FITC in naïve recipients [58, 167]. Subsequently, we and others demonstrated that the depletion of CD301b+ cDC2s in various organs of the *Mgl2*‐DTR mice results in significantly impaired Th2 differentiation of antigen‐specific CD4 T cells without mitigating their differentiation into Th1 or Th17 cells in the skin‐dLN, lung and lung‐dLN following exposure to type 2 adjuvants (papain, alum), helminth parasites (*Nippostrongylus brasiliensis*), or fungal spores (*A. fumigatus*) [60, 150, 170, 171].

Upon intradermal infection with *N. brasiliensis*, in addition to migratory CD301b+ cDC2s, Ly6C+ mo‐DCs also efficiently take up the parasite antigen [172]. However, while CD301b+ cDC2s isolated from the *N. brasiliensis*‐infected mice effectively induce IL‐4‐producing Th2 cells when adoptively transferred into naïve recipients, the mo‐DCs do not show this capacity [172]. Likewise, in mice intratracheally administered with house dust mite allergen, both CD11b+ cDC2s and mo‐DC‐like cells marked by CD64 and the MAR‐1 mAb in the lung efficiently take up the antigen, but the latter population contributes minimally to antigen‐bearing migratory DCs in the lung‐dLN and is much less efficient in priming Th2 cells. These mo‐DC‐like cells, however, produce higher levels of chemokines that act on eosinophils and monocytes compared to the CD11b+ cDC2s, suggesting their role in inflammation [173]. Originally identified as mo‐DCs, these mo‐DC‐like cells were later found to be of pre‐cDC origin and reclassified as inflammatory cDC2s, which universally express CD64 and partially express Ly6C along with other cDC2 markers such as PD‐L2 but are negative for CD301b [49]. In agreement with the data in the *Mgl2*‐DTR mice, these studies suggest that the Th2 priming capacity resides in a selective subset of cDC2s [49, 173].

Notably, targeting exogenous antigens to CD301b+ cDC2s in vivo using an anti‐CD301b monoclonal antibody (mAb) induces a Th2‐biased immune response [167], suggesting that CD301b+ cDC2s are not only required but also sufficient for Th2 cell differentiation. Indeed, experiments in mice expressing a Cre‐removable DTR cassette in CD11c+ cells (CD11c‐dlDTR) crossed with the *Mgl2*‐Cre strain (*Mgl2*‐Cre; CD11c‐dlDTR mice), in which CD301b+ cDC2s are protected from DT‐induced depletion while all CD301b− cDCs and other CD11c+ cells remain susceptible, demonstrated normal Th2 cell differentiation despite a significant reduction in the total cDC numbers, indicating the sufficiency of CD301b+ DCs for driving Th2 cell differentiation even in the absence of other cDC subsets [150]. The reduced Th2 cell differentiation in the *Mgl2*‐DTR mice is consistent with other mouse models lacking cDC2 subsets due to the conditional deletion of *Irf4* or *Klf4* or the mutation in the enhancer loci in the *Zeb2* gene [48, 52, 55, 154]. However, since CD301b is expressed only in a subpopulation of cDC2B cells [29, 56, 57], CD301b+ cDC2s appear to represent a minimal cDC2 subset in mice so far identified to be specifically required and sufficient for inducing Th2 cell differentiation.

### 4.4 Regulation of CD4+ T Cell Priming Dynamics by CD301b+ cDC2s

In the skin, the dermal CD301b+ cDC2s represent the majority of migratory APCs that migrate to the dLN within 24 h upon exposure to immunological insults, remarkably faster than the migration kinetics of the epidermal LCs and the dermal cDC1s which often take more than 2 days to reach the dLN [58, 168, 174, 175]. As such, depleting CD301b+ cells in the Mgl2‐DTR reduces migratory APCs in the skin–dLNs by 60%–70% and nearly completely eliminates hapten‐carrying cells in the dLN 24 h after hapten painting on the skin in the contact hypersensitivity model [60, 169]. In the skin–dLNs, CD301b+ cDC2s are localized to the T‐B border and segregated from cDC1s and LCs as mentioned above, but they are also segregated from CD301b− CD172a+ cDC2s and CD301b− CD172a− cDC2s, which reside in the lower cortical ridge and the distal regions near the cortico‐medullary cords, respectively, indicating territorial compartmentalization among cDC2 subsets in the LNs [176].

**FIGURE 1:**
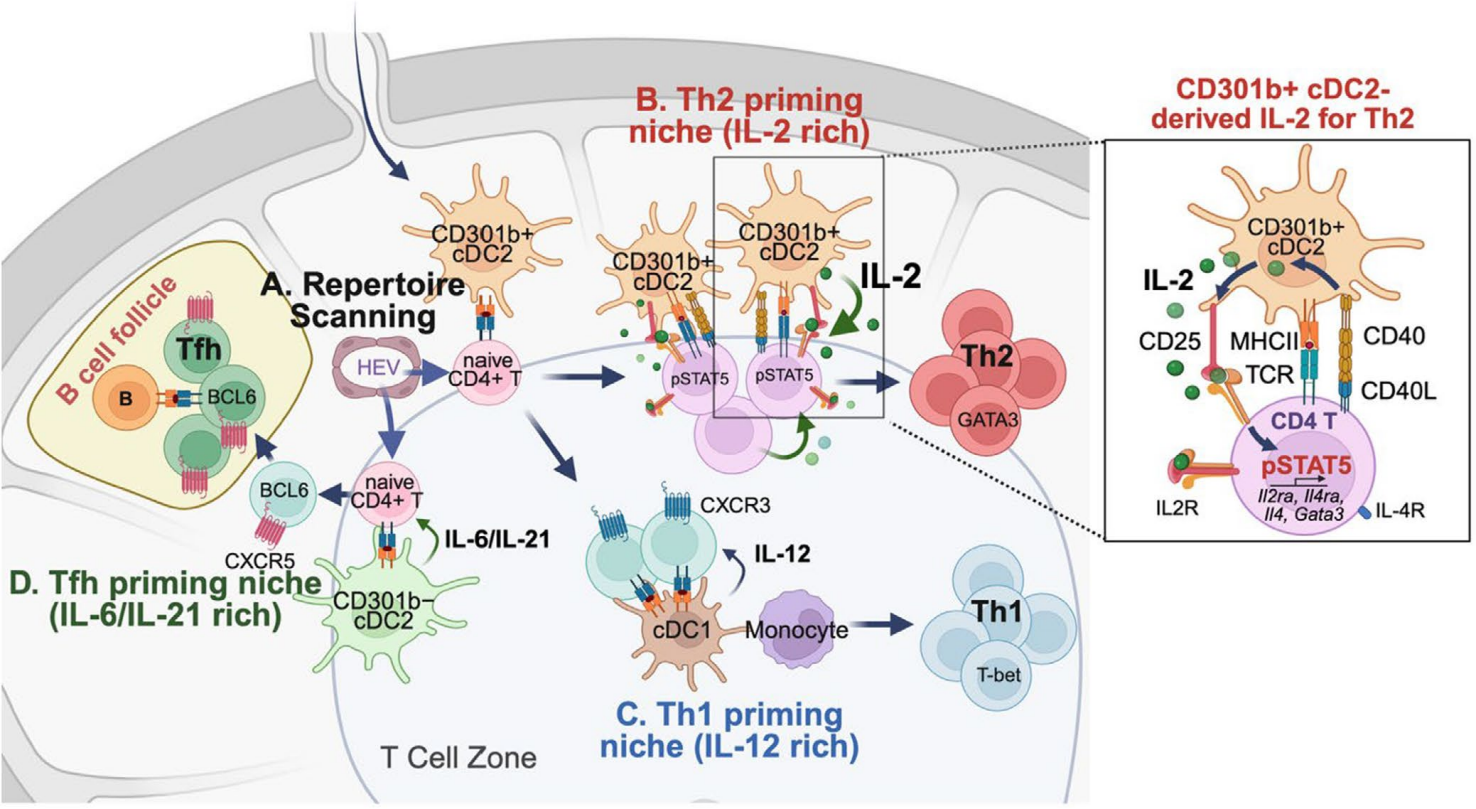
Division of labor between cDC subsets in Th cell differentiation. (A) CD301b+ cDC2s efficiently engage incoming naïve polyclonal CD4+ T cells near the HEVs at the T‐B border, facilitating repertoire scanning to maximize the expansion of cognate clones in both Th1‐ and Th2‐biased contexts. (B) Under Th2‐biased conditions, CD301b+ cDC2s trigger the formation of stable Th2 macro‐clusters and, upon CD40 ligation, enhances their intrinsic IL‐2 production that drives maximal IL‐2R signaling in the antigen specific CD4+ T cells, a critical signal for the Th2 fate commitment. CD301b+ cDC2‐intrinsic CD25 expression facilitates the directed IL‐2 delivery to the cognate CD4+ T cells by preventing it from being consumed by surrounding Tregs, thereby promoting Th2 differentiation while suppressing the Tfh cell fate. (C) In contrast, under Th1‐biased conditions, the activated CD4+ T cells upregulate CXCR3 and migrate to the T cell zone, where they encounter IL‐12‐producing cDC1s and inflammatory monocytes that home to the T cell zone and promote Th1 cell differentiation. (D) CD301b+ and CD301b− cDC2s are spatially segregated and promote Th2 and Tfh differentiation, respectively. CD301b− cDC2s prime CD4+ T cells to upregulate CXCR5, which guide them toward the B cell follicles where they differentiate into Tfh cells. Created with BioRender.com.

Within the T‐B border niche in the LN, CD301b+ cDC2s are especially enriched in the areas surrounding the HEVs, where they retain the incoming pool of naïve polyclonal CD4+ T cells by an MHCII‐dependent but antigen‐independent mechanism and scan their TCR repertoire (Figure 1A) [169]. Experiments in mice immunized with OVA and adoptively transferred with OVA‐specific TCR transgenic OT‐II CD4+ T cells showed that CD301b+ cDC2s preferentially form physical conjugates with OT‐II cells within 3 h of OT‐II cell entry into the dLNs. This process is compromised by pharmacological displacement of CD301b+ cDC2s from the areas immediately surrounding HEVs, indicating that the strategic positioning of CD301b+ cDC2s around the HEVs directly contributes to their functional specialization in T cell priming [169]. While CD301b+ cDC2s are required specifically for Th2 cell differentiation, the repertoire scanning by CD301b+ cDC2s is a common process under diverse conditions, including immunization with papain (a Th2‐biased adjuvant), Freund's complete adjuvant (FCA, a Th1/Th2‐mixed adjuvant), or with CpG DNA (a Th1‐biased adjuvant), indicating that CD301b+ cDC2s engage naïve CD4+ T cells regardless of the type of immune stimulus [60, 169]. Consequently, depletion of CD301b+ cDC2s significantly delays the priming of OT‐II cells in mice immunized with OVA with either type 1 or type 2 adjuvants. In the absence of CD301b+ DCs, the likelihood of productive OT‐II cell expansion is markedly reduced when the input number of donor OT‐II cells is low, highlighting the critical role of CD301b+ DC2s and their specific positioning in the LN in effectively priming rare antigen‐specific CD4+ T cell clones (Figure 1A) [169, 177].

### 4.5 Molecular Basis for the Specific Requirement of CD301b+ cDC2s for Th2 Cell Differentiation

Unlike the induction of other Th cell fates such as Th1 and Th17 that are typically induced by cDC‐derived cytokines like IL‐12 and IL‐6/IL‐23, respectively [73], the DC‐derived factors that directly act on CD4 T cells to imprint the Th2 cell fate remain elusive. In contrast to the sufficiency of CD301b+ cDC2s for Th2 cell differentiation demonstrated by antigen targeting with anti‐CD301b mAb and depletion of CD301b− DCs in vivo [150, 167], the sort‐purified CD301b+ cDC2s pulsed with antigens in vitro have thus far failed to induce Th2 cell differentiation [60, 167]. Since the Th2 fate is preferentially induced by intermediate levels of TCR signal intensity rather than by strong TCR stimulation [178], the priming dynamics appear to be tightly controlled for optimal Th2 cell priming, which may not be fully recapitulated simply by loading antigens on CD301b+ cDC2s in vitro. Nevertheless, CD301b+ cDC2‐intrinsic expression of MHCII and CD40 is required for Th2 cell differentiation [150], suggesting that CD301b+ cDC2s instruct the Th2 fate through cognate interaction. Precise analysis of the OT‐II cell priming dynamics in mice depleted of CD301b+ cDC2s found a partial but significant reduction in antigen‐induced upregulation of CD25, the high affinity subunit of IL‐2 receptor (IL‐2R), in the OT‐II cells throughout the course of their priming [150]. Notably, the competitive transfer of OT‐II cells with partially reduced (*Il2ra* ^*+/*−^) and intact (*Il2ra* ^*+/+*^) CD25 expression into WT recipients immunized with OVA plus papain, FCA, or CpG DNA results in a severe reduction of Th2 differentiation without overtly impacting Th1 differentiation in the *Il2ra* ^*+/*−^ OT‐II cells compared to their *Il2ra* ^*+/+*^ counterparts, indicating that Th2 differentiation is more stringently dependent on the IL‐2R signaling than Th1 differentiation. Thus, while IL‐2 is ultimately required for the differentiation of both Th1 and Th2 cells [123, 126, 134, 137, 179, 180, 181, 182, 183, 184, 185], the partial reduction of CD25 in OT‐II cells primed in mice lacking CD301b+ cDC2s can account for the loss of Th2 cell differentiation in those mice [150].

If Th2 differentiation is more sensitive to the IL‐2 dosage than Th1 cells, the IL‐2 availability needs to be ensured during Th2 cell priming. However, Th2 differentiation is generally favored by weak TCR stimulation while strong signals tend to promote Th1 differentiation, yet the level of IL‐2 production generally correlates with the TCR signal strength [186, 187]. In addition, IL‐2 produced by activated T cells in the LNs is rapidly sequestered by the surrounding Tregs [132, 133, 188], all of which implies the presence of an active mechanism to guarantee the availability of IL‐2 for the cognate CD4+ T cell clones destined to become Th2 cells. Consistently, a few recent studies demonstrated the importance of localized IL‐2 availability within T cell clusters formed during Th2 cell differentiation [180, 189, 190, 191]. These Th2‐initiating T cell “macro‐clusters” are characterized by dense aggregation of proliferating Th2 cells at the T‐B border areas in the dLNs, and their formation requires interactions with surrounding cDC2s, likely CD301b+ cDC2s, as these clusters are significantly reduced in mice lacking IRF4 in CD11c+ cells (Figure 1B) [189]. However, while the formation of activated T cells may ensure exposure to paracrine IL‐2 from neighboring T cells, a single antigen‐specific CD4+ T cell alone would not be able to secure IL‐2 availability through this mechanism until it proliferates to a certain level enough to form a cluster. Puzzlingly, however, in mice transferred with OT‐II cells and immunized with OVA plus papain, the expression of CD25 in OT‐II cells peaks at around 16 h after the priming, well before the initial cell division of activated OT‐II cells [150, 169], suggesting the presence of an additional source of IL‐2 early after priming. Indeed, CD40 stimulation induces IL‐2 production and upregulates CD25 expression specifically in CD301b+ cDC2s in vivo. Importantly, genetic deletion of MHCII, IL‐2, or CD25 specifically in CD301b+ cDC2s results in reduced CD25–STAT5 signaling and impaired Th2 differentiation in the donor OT‐II cells without affecting their Th1 differentiation but instead increasing the Bcl6+ PD‐1+ Tfh precursors [150]. Thus, the IL‐2 production by CD301b+ cDC is crucial for maximizing the early IL‐2R signaling in the cognate CD4+ T cells, whereas CD301b+ cDC2‐intrinsic CD25 expression facilitates directed IL‐2 delivery to the cognate CD4+ T cells by preventing it from being consumed by surrounding Tregs, which both facilitates Th2 differentiation while suppressing the Tfh cell fate (Figure 1B) [150].

## 5 Division of Labor Between cDC2 Subsets

### 5.1 Division of Labor Between CD301b+ cDC2s and CD301b− cDCs in Th1 Differentiation

The strategic positioning of CD301b+ cDC2s near the HEVs at the T‐B border areas in the LN remains unchanged regardless of the immunization status, allowing them to efficiently engage incoming naïve polyclonal CD4+ T cells for efficient repertoire scanning in both Th1‐ and Th2‐biased contexts (Figure 1A) [169]. However, the depletion of CD301b+ cDC2s does not cause an overt loss of Th1 cells but rather results in an increase in the relative abundance of Th1 and Th17 cells at the clonal level when the mice are immunized with a mixed adjuvant, likely representing a clonal shift from Th2 to Th1/Th17 cells [169]. Unlike Th2 cells that form stable macro‐clusters with CD301b+ cDC2s near the T‐B border, Th1 cells in mice immunized with OVA plus CpG DNA are scattered in smaller clusters throughout the T cell zone and outer paracortex of the dLN, where IL‐12‐producing antigen‐bearing migratory cDC1s are localized [189]. In addition, the inflammation typically induced by such Th1‐biased adjuvants recruits an influx of IL‐12‐producing monocytes that enter the dLN through the HEVs and position within the T cell zone to support Th1 cell differentiation [83], a process not elicited under Th2‐biased conditions [83, 189, 192]. Thus, while CD301b+ cDC2s may still be the first cDC population to present antigens to Th1 precursors upon their dLN entry, the Th1 fate decision appears to be dictated more predominantly by the additional interaction with other APC subsets that supply IL‐12 (Figure 1C) [177]. Intriguingly, the chemokine receptor CXCR3 is rapidly upregulated in antigen‐specific CD4+ T cells upon immunization with Th1‐biased adjuvants while cDC1s and monocytes express its ligands CXCL9 and CXCL10 [193, 194], the latter of which facilitates CD4+ T cell–DC interaction and paracortical retention of Th1 cells [193, 195]. Importantly, both *Cxcr3* ^*−/−*^ CD4+ T cells and *Cxcl10* ^*−/−*^ cDCs show impaired Th1‐inducing potential [193]. Together, these observations suggest a sequential priming model for Th1 differentiation in vivo, in which initial activation by CD301b+ cDC2s at the T‐B border near HEVs, followed by secondary instruction by IL‐12‐producing cDC1s and/or monocytes in the T cell zone (Figure 1C) [177].

Despite the high capacity of cDC1s in antigen cross‐presentation and Th1 induction, cDC2s, not cDC1s, are the primary APCs that induce initial CD4T cell priming or even their Th1 differentiation upon infection with vaccinia virus (VV) or lymphocytic choriomeningitis virus (LCMV), respectively [196, 197]. However, cDC1s later function as a crucial platform for delivering CD4+ T cell help by forming co‐clusters with antigen‐specific CD4+ and CD8+ T cells [197]. Likewise, upon epidermal infection with herpes simplex virus (HSV)1, which causes more localized infection than VV or LCMV, migratory cDC2s prime virus‐specific CD4+ T cells within 12 h of infection, while LN‐resident cDC1s prime CD8+ T cells 1–2 days post‐infection and co‐cluster with CD4+ T cells delivering help for CD8+ T cells [198]. Importantly, cDC1s are dispensable for the HSV1‐induced CD4+ T cell expansion but required for their full Th1 differentiation [199], and the CD8+ T cell priming by cDC1s requires migratory cDC2s [174], which potentially include CD301b+ cDC2s. Thus, collaboration between multiple cDC2 and cDC1 subsets may be a common mechanism for Th1 and CD8+ T cell priming in vivo across different conditions of type 1 immunity.

### 5.2 Division of Labor Between cDC2 Subsets in Tfh–Non‐Tfh Bifurcation

Production of antigen‐specific antibodies is fundamental to mammalian adaptive immunity and is induced universally across different types of immune responses. Tfh cells provide critical help for the differentiation of GCB cells, the source of cells producing high‐affinity, class‐switched antibodies. Thus, concomitant development of Tfh and non‐Tfh Th1, Th2, or Th17 effector cells specific for the same antigen is also universal across all types of adaptive immunity. Accordingly, various antigen delivery strategies targeting specific cDC subsets have demonstrated the ability of multiple cDC subsets to induce antigen‐specific humoral responses regardless of the cDC subset targeted [200, 201, 202]. Puzzlingly but consistently, the depletion of cDC1s and/or LCs in the *Batf3* ^*−/−*^, *Xcr1*‐DTR, or in *Cd207*‐DTR mice does not overtly affect the development of Tfh or GC B cells against general antigens unless the antigens are targeted specifically to these depleted APC subsets [50, 59, 156, 203, 204, 205], indicating that any remaining APCs are generally *sufficient* to induce Tfh cells, potentially with an altered flavor to the concomitantly developing non‐Tfh effector Th cells. Nevertheless, the depletion of pan‐DCs in the CD11c‐DTR abrogates Tfh differentiation when the antigen dose is low, whereas a high‐dose antigen permits compensatory B cell‐driven Tfh development [206], suggesting that cDCs are generally required to initiate Tfh differentiation at physiological antigen concentrations [207]. Furthermore, in the CD11c/βb mice, in which MHCII expression is restricted to CD11c+ cells, the cDC‐restricted MHCII expression is sufficient to skew naive CD4 T cells toward Tfh cell precursors, whereas the subsequent interaction with non‐cDC APCs, presumably B cells, is necessary for the maximal expression of the Tfh signature molecules such as PD‐1 and IL‐21 [208]. These studies suggest stepwise fixation of the mature Tfh phenotype through sequential interactions with cDCs and B cells.

While the absence of cDC1 and/or LCs does not generally affect Tfh differentiation, mice lacking cDC2s or their subset(s) are often associated with impaired Tfh differentiation and/or reduced antibody production [127]. For example, mice with *Zeb2* enhancer mutations or those lacking IRF4 in CD11c+ cells show significantly reduced antigen‐specific IgG1 and IgE upon strictly enteric helminth infection or subcutaneous immunization with papain, respectively [48, 52]. Likewise, mice lacking KLF4 in CD11c+ cells and thereby lacking some cDC2B subsets show reduced IgG1 production upon subcutaneous immunization with Schistosoma egg antigen [55], whereas mice lacking cDC2A subsets due to the lack of NOTCH2 in CD11c+ cells show reduced Tfh and GCB cell differentiation when immunized intravenously with sheep erythrocytes [156], which is also consistent with the diminished antibody production upon intravenous immunization with allogenic erythrocytes in mice with CD11c+ cell‐specific IRF4 deficiency [209]. The requirement of cDC2A subsets for Tfh induction is also supported in mice with DC‐specific deletion of *Bcl6*, which results in a specific reduction of NOTCH2‐dependent cDC2A subsets as well as partially impaired Tfh differentiation and antibody titers upon intraperitoneal immunization with OVA plus poly(I:C) or alum, though this model appears to show more significant reduction in T follicular regulatory (Tfr) cells than in Tfh cells [157]. The role of cDC2s in Tfh differentiation is not specific to type 2 immune responses, as Krishnaswamy et al. demonstrated that mice with cDC2‐specific migration defect due to the deletion of *Dock8* in CD11c+ cells show reduced Tfh cell development following subcutaneous or intranasal immunization with OVA and LPS or with nanoparticle‐formulated vaccine adjuvant PLGA, as well as diminished production of antiviral IgG upon intranasal infection with influenza virus [50]. Furthermore, human tonsillar CD1c (BDCA1)+ cDC2s, compared to other APC populations such as pDCs, CD141 (BDCA3)+ cDC1s and CD14+ macrophages, preferentially induce IL‐21‐producing CXCR5+ PD‐1+ Tfh cells in vitro, highlighting a conserved role of cDC2s in human Tfh differentiation [210]. Mechanistically, Li et al. proposed that cDC2 cells augment Tfh cell differentiation by producing soluble CD25, which then quenches IL‐2 and limits its availability to CD4+ T cells, thereby unleashing the Tfh precursors from the IL‐2R‐mediated suppression [204]. Thus, although the responsible subsets may be different in different models, these studies collectively indicate the requirement of cDC2s or their subset(s) for efficient differentiation of Tfh cells.

Interestingly, however, selective depletion of CD301b+ cDC2s in the *Mgl2*‐DTR mice enhances the development of Tfh cells, GC B cells, and antibody production without affecting Tfr cells upon subcutaneous immunization with the protein antigen OVA with papain, LPS, CpG DNA, or even without adjuvant, as well as upon intraperitoneal immunization with OVA and alum or epicutaneous exposure to the whole extracts from house dust mite [59]. Moreover, continuous depletion of CD301b+ cDC2s alone, without active immunization, results in enhanced development of self‐reactive antibodies [59]. The over‐expansion of Tfh occurs only when CD301b+ cDC2s are depleted within 2 days of immunization, indicating their suppressive role during early priming. The enhanced Tfh differentiation in these mice is presumably due to the loss of PD‐L1 that is highly expressed in CD301b+ cDC2s, as the Tfh expansion induced by PD‐L1 blockade in WT mice is not further exaggerated in mice depleted of CD301b+ cDC2s [59]. Notably, mice lacking IL‐2 specifically in CD301b+ cDC2s also exhibit accumulation of immature Tfh cells in the dLN; though, unlike the *Mgl2*‐DTR mice, the number of mature Tfh cells is under control in this model, likely because the PD‐L1‐dependent regulatory mechanism still remains intact in those mice [150].

Collectively, these findings highlight a division of labor between CD301b^−^ cDC2s and CD301b+ cDC2s to promote Tfh and Th2 differentiation, respectively (Figure 1B,D). In agreement, while the initial phase of Tfh cell priming occurs predominantly at the T‐B border and interfollicular region [67], CD4+ T cells within the Th2 macro‐clusters surrounded by CD301b+ DCs rarely include Tfh cells [189]. Moreover, not all activated CD69+ CD4 T cells upregulate CD25 to high levels [124], a process required specifically for the Th2 fate decision and dependent on IL‐2 production by CD301b+ cDC2s [150]. Given the segregation between different cDC2 subsets in the LN [176], these observations support a model in which non‐Tfh and Tfh priming are spatially segregated events occurring in parallel in two distinct microenvironments, an IL‐2‐rich niche shaped by CD301b+ cDC2s favoring Th2 differentiation and a pro‐Tfh niche that is supported by CD301b− cDC2s and potentially enriched in Tfh‐supporting cytokines such as IL‐6 and IL‐21 (Figure 1B,D). In addition to the spatial segregation between cDC2 subsets, temporal differences in cDC2 exposure to type I IFN (IFN‐I) may also shape their ability to instruct Tfh differentiation in type 1 immune responses following a viral infection, as De Giovanni et al. demonstrated that exposure to IFN‐I early during infection with recombinant vesicular stomatitis virus (VSV) induces IL‐6 production from migratory cDC2s and mo‐DCs and promotes Tfh cell priming, whereas late IFN‐I exposure during infection with LCMV is associated with low IL‐6 production and favors non‐Tfh (Th1) differentiation [196]. However, an alternative model is also possible, whereby all CD4+ T cells are initially primed by CD301b+ cDC2s to first acquire a pre‐Th2 state but later redirected toward the Tfh lineage through subsequent interaction with CD301b− cDC2s. Dissecting these possibilities will require further investigation.

### 5.3 Division of Labor Between cDC2 Subsets in Th17 Differentiation

In addition to the defective Th2 priming, earlier studies on mice lacking IRF4 in CD11c+ cells found a significant reduction in Th17 cells in the small intestine, lung, and their respective dLNs, which was associated with selective loss of CD11b+ CD103+ cDC2s and CD11b+ cDC2s in the intestine and lung, respectively [43, 51]. This is reminiscent of the selective loss of intestinal CD11b+ CD103+ cDC2s in the mice with conditional deletion of *Notch2* in CD11c+ cells, which is also associated with impaired Th17 differentiation that causes increased susceptibility to *C. rodentium* infection [53, 54]. Furthermore, the reduced Th17 differentiation and increased *C. rodentium* susceptibility are also associated with the reduced frequency of intestinal CD11b+ CD103+ cDC2s in mice with cDC‐specific deletion of *Bcl6* [157]. Notably, Welty et al. demonstrated intact Th17 differentiation upon specific deletion of MHCII on the intestinal CD103+ CD11b+ cDC2s in the MHCII conditional knockout mice driven by the Cre recombinase regulated by the human *CD207* promoter, indicating that direct antigen presentation by CD103+ CD11b+ cDC2s is dispensable for the development of Th17 cells [211]. In contrast, mice lacking *Klf4* in CD11c+ cells show a mild reduction of intestinal CD172a+ cDC2s but do not show altered susceptibility to *C. rodentium*, suggesting intact Th17 differentiation in the intestine [55]. These studies suggest the division of labor between the NOTCH2‐dependent CD11b+ CD103+ cDC2s and KLF4‐dependent CD11b+ CD103− cDC2s for inducing Th17 and Th2 differentiation, respectively. Given the selective expression of CD301b in a subset of NOTCH2‐independent cDC2s across different organs [29, 56, 57], CD301b+ cDC2s are not likely required for Th17 differentiation at least in the intestine, though their role in the gut has not been directly examined.

The co‐expression of CD11b and CD103 marks a discrete population of cDCs in mouse intestine and mesenteric LNs that disappears in mice lacking IRF4 or NOTCH2 in CD11c+ cells [43, 51, 53, 212]. However, like the NOTCH2‐dependent ESAM hi cDC2A in the spleen, this population is unique to the intestine and the expression of CD103 marks a subset of CD11b− cDC1 cells in most other peripheral organs [16]. In fact, mice lacking NOTCH2 in CD11c+ cells have normal numbers of CD11b+ cDC2s, CD103+ cDC1s, and epidermal LCs in the skin, liver, lung and their respective dLNs except for the reduction of a minor subset of LN‐resident cDC2s that express ESAM, CD11b and CD4 [53, 54], though the analysis of mice lacking the NOTCH signaling component RBPJ in cDCs showed significant reduction of T‐bet+ cDC2As in the lung and liver [57]. Accordingly, the mice with CD11c+ cell‐specific *Notch2* deletion show no impact on lung Th17 cells when intranasally infected with *S. pyogenes* [155]. In contrast, mice lacking IL‐6 specifically in CD301b+ cDC2s exhibit impaired Th17 differentiation in this model, suggesting the dual role of CD301b+ cDC2s in promoting Th2 and Th17 cells in the lung [155]. However, the requirement of the lung CD301b+ cDC2s for Th17 cell differentiation may be model‐dependent, as they have been shown to preferentially induce Th2 over Th17 cells in the models of *A. fumigatus* and dust mite extract exposure [170, 213].

Like in the lung, the role of cDC‐intrinsic *Notch2* remains unclear in the skin. However, the depletion of epidermal LCs, but not dermal CD103+ CD207+ cDC1s or CD301b+ cDC2s, results in a specific reduction of Th17 cells upon infection with *Candida albicans* in mice [80, 214]. LCs are developmentally related to tissue resident macrophages, but they are phenotypically similar to migratory cDCs with high migration and antigen presentation capacity and account for most of the migratory APCs in the epidermis. Importantly, LC‐intrinsic MHCII expression is both required and sufficient, and LC‐derived IL‐6 is required for the *C. albicans*‐induced Th17 differentiation in the skin‐dLN, though they are not required for the overall expansion of antigen‐specific CD4+ T cells [80, 214]. Notably, upon subcutaneous immunization with OVA and FCA, the depletion of CD301b+ DCs in the *Mgl2*‐DTR mice leads to a loss of Th2 cells with a reciprocal increase in Th1 and Th17 cells in the skin‐dLNs [169], suggesting that CD301b+ DCs promote the Th2 fate at the cost of Th1/Th17 differentiation at the clonal level. Like the Tfh–non‐Tfh bifurcation, the bifurcation between the Th2 and Th1/Th17 may potentially be mediated by the IL‐2 produced from the CD301b+ cDC2s, as IL‐2 is known to directly suppress Th17 polarization [138, 139]. These findings suggest a division of labor between CD301b+ cDC2s and LCs, with the former favoring Th2 polarization and the latter promoting Th17 cell differentiation in the skin.

**FIGURE 2:**
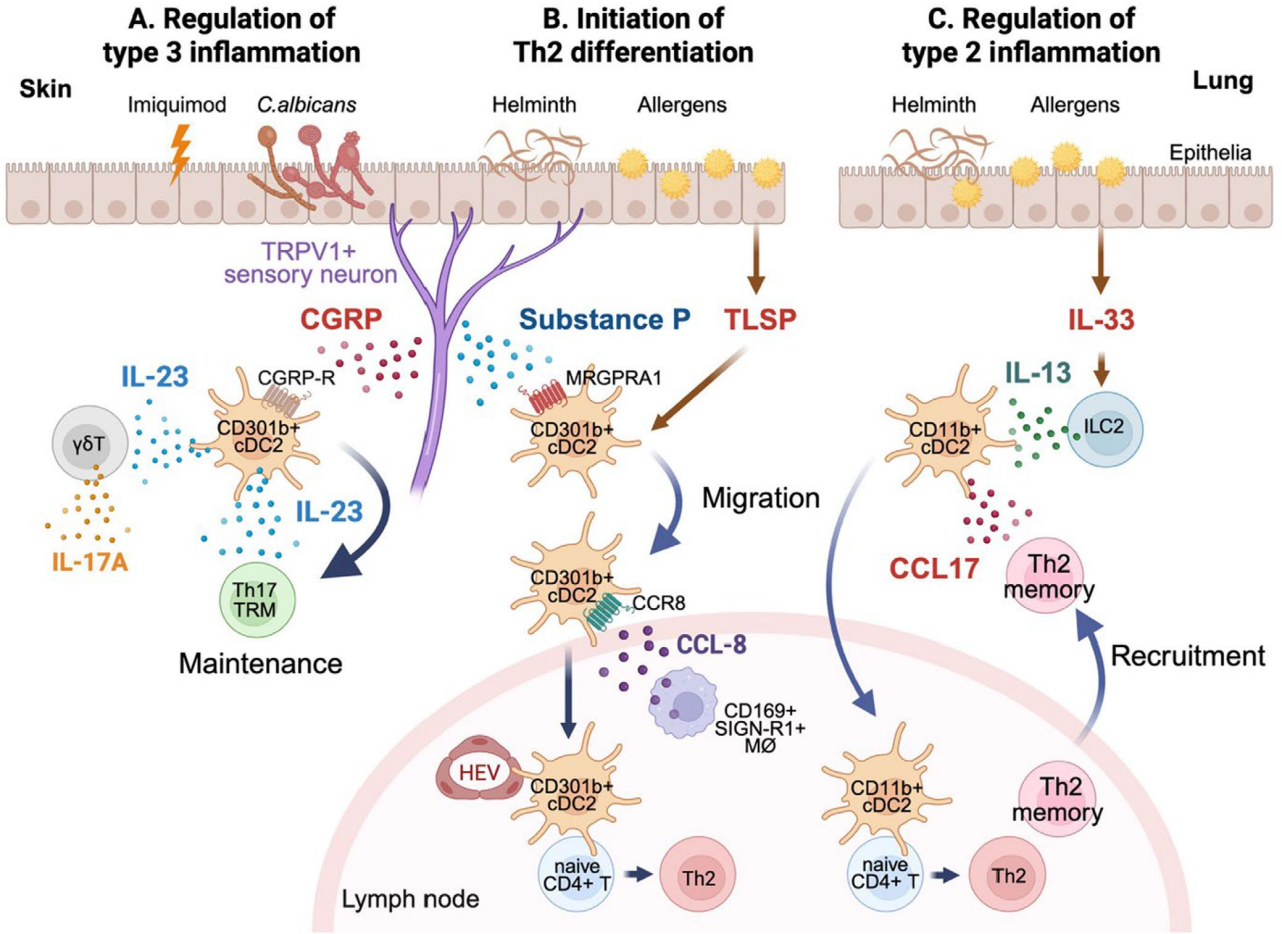
Regulation of cDC2 subsets by tissue‐derived cues. (A) In *C. albicans* infection or imiquimod‐induced psoriasis in the skin, the neuropeptide CGRP produced by TRPV1+ sensory neurons triggers the production of IL‐23 by dermal CD301b+ cDC2s via the CGRP receptor (CGRP‐R), which in turn induces IL‐17A production by dermal γδT cells and supports the maintenance of tissue‐resident memory Th17 (Th17 TRM) cells in the skin. (B) Upon Th2‐biased immunizations, such as allergen or helminth exposure, dermal CD301b+ cDC2s are activated and migrate to the dLNs. In mice immunized with a protease allergen papain, the migration of CD301b+ cDC2s is triggered by TRPV1+ sensory neuron‐derived substance P (SP) through the receptor MRGPRA1 on CD301b+ cDC2s. Additionally, certain contact allergens induces TSLP in the skin, which also mobilizes dermal cDC2s to the dLN. The production of CCL8 from the CD169+ SIGN‐R1+ interfollicular MØs in the dLN in response to the papain exposure directs the entry of CD301b+ cDC2s into the LN parenchyma via the CCR8 signaling. Once CD301b+ cDC2s reach the LN parenchyma, they are preferentially positioned in the outer T cell zones near the T‐B border, where HEVs are densely distributed. (C) Upon proteolytic damage, IL‐33 released from the epithelial cells stimulates ILC2s to produce IL‐13, which then promotes the migration of CD103− CD11b+ cDC2s to the dLN and licenses them to produce the Th2‐attracting chemokine CCL17. This chemokine recruits memory Th2 cells to the peripheral tissue upon allergen re‐exposure. Created with BioRender.com.

Interestingly, however, while CD301b+ cDC2s are dispensable for the *C. albicans*‐induced Th17 differentiation, they are nevertheless required for the induction of IL‐17A from the dermal γδT cells in the same infection model, as well as in the model of imiquimod‐induced psoriasis. In these models, CD301b+ cDC2s serve as a major source of IL‐23, a potent inducer of IL‐17, in response to calcitonin gene‐related peptide (CGRP) released from the sensory neurons (Figure 2A) [61, 215, 216]. Moreover, even though the priming of *C. albicans*‐specific Th17 cells does not require CD301b+ cDC2, their IL‐23 production is crucial for maintaining tissue‐resident memory Th17 cells in the *C. albicans*‐infected skin (Figure 2A) [217]. Thus, CD301b+ cDC2s can both negatively and positively regulate Th17 cells through clonal skewing and IL‐6/IL‐23 production, respectively, but these studies seem to suggest the general importance of CD301b+ cDC2s in type 3 immunity.

In summary, while the division of labor between developmentally distinct cDC subsets appears to drive Th differentiation in general, CD301b+ cDC2s in different organs exhibit mixed and model‐dependent Th17‐inducing potential, possibly reflecting their functional plasticity. Evidence suggests that cDC2s can acquire distinct T cell‐priming abilities depending on environmental stimuli, highlighting their functional plasticity. For example, elevated cyclic AMP levels in the environment or stimulation with curdlan, a microbial Dectin‐1 agonist, reprograms cDC2s from a pro‐Th2 to a pro‐Th17 phenotype, which correlates with downregulation of *Irf4* and *Klf4* and upregulation of *Il23a* in cDC2s [218]. Likewise, the Th2‐polarizing program in human mo‐DCs can be overridden by environmental cues, such as simultaneous exposure to IL‐12 and IL‐1, to induce a pro‐Th17 phenotype [219]. Alternatively, CD301b+ cDC2s may be comprised of two distinct subpopulations with different Th2‐ and Th17‐inducing potential, which need to be further dissected.

### 5.4 Division of Labor Between cDC2 Subsets in Immunoregulatory Responses

Induction of pTregs is crucial for maintaining tissue homeostasis especially in barrier organs that are constantly exposed to commensal microbes [220, 221, 222, 223]. Migratory cDCs play an essential role in the induction, retention and activation of pTregs [224, 225]. Several studies have agreed upon the higher tolerogenic potential of cDC1s than cDC2s [226, 227, 228, 229, 230, 231, 232, 233]. However, the tolerogenic potential of cDCs may be determined by the environmental cues rather than by the developmental origin, as tumor antigen uptake renders both cDC1s and cDC2s tolerogenic through the induction of a conserved regulatory program [234]. Interestingly, Gargaro et al. demonstrated that, while IDO1, the rate‐limiting enzyme for the conversion of tryptophan to immunoregulatory kynurenines, is selectively expressed by cDC1s at the steady state, the cDC1‐derived L‐kynurenine induces IDO1 expression in cDC2s and makes them tolerogenic during inflammation in an aryl hydrocarbon receptor (AhR)‐dependent manner, suggesting synergistic operation between cDC1s and cDC2s [235]. Accordingly, an earlier study by Welty et al. demonstrated a significant reduction of gut‐homing Tregs in mice co‐depleted of CD11b− CD103+ cDC1s and CD11b+ CD103+ cDC2As at the steady state, but not in those with individual depletion [211].

Nevertheless, the tolerogenic potential of cDCs has been identified specifically in cDC2s or their subset in certain models [236, 237, 238, 239]. For instance, while mesenteric LN cDC1s are more efficient than their cDC2 counterparts in inducing the pTreg conversion of TCR‐stimulated CD4+ T cells in vitro [229, 230], a study with commensal‐specific TCR transgenic CD4+ T cells found impaired commensal‐specific pTreg induction in the mesenteric LNs in the mice lacking cDC2A cells due to DC‐specific *Notch2* deficiency, but not in the cDC1‐null *Batf3* ^*−/−*^ mice [237]. In the neonatal mouse skin, Weckel et al. demonstrated that CD301b+ cDC2s specifically take up commensal antigens, acquire maturation programs, and express tolerogenic markers including the *Aldh1a2*, which encodes the retinoic acid‐producing enzyme RALDH2, facilitating the generation of commensal‐specific pTreg cells in an MHCII‐dependent manner [240]. Likewise, upon topical application of OVA protein without adjuvant in adult mouse skin, CD11b+ PD‐L2+ cDC2s, a major part of which is CD301b+ cDC2s, but not PD‐L2− cDCs capture the antigen and expand OVA‐specific pTregs ex vivo [241]. Furthermore, in a mouse model of atopic dermatitis induced by a repeated topical exposure to a vitamin D3 analog MC903, the induction of TSLP, the same cytokine responsible for CD301b+ cDC2 migration in FITC‐induced contact hypersensitivity, by MC903 has been shown to trigger pTreg expansion by mobilizing cDC2 subsets to the dLN [242, 243], suggesting context‐dependent tolerogenicity of CD301b+ cDC2s.

In the steady‐state mouse lung, CD64− (i.e., non‐monocytic) cDC2s can be separated into two populations based on the expression of TNF receptor 2 (TNFR2). The TNFR2− population is largely CD301b−, whereas the TNFR2+ cDC2s consist of CD301b− and CD301b+ cells, the latter of which specifically express high levels of IDO1 in naïve mice [93, 244]. In addition to the loss of cDC2s and allergen‐ and helminth‐induced Th2 cell differentiation, mice lacking IRF4 in CD11c+ cells develop spontaneous CD4+ T cell expansion with loss of endogenous and antigen‐induced pTregs in the lung [93]. Importantly, reconstituting those mice with CD301b+ TNFR2+ cDC2s, but not CD301b− TNFR2+ cDC2s or TNFR2− cDC2s, restores the number of pTregs in an interferon‐α receptor 1 (IFNAR1)‐dependent manner, as IFN‐I induces IDO1 expression specifically in TNFR2+ cDC2s [93]. Moreover, exposure to house dust mite allergen reduces their IFNAR1 expression and switches the phenotype of TNFR2+ cDC2s from tolerogenic to Th2‐immunogenic, while treating allergen‐exposed mice with exogenous IFN‐I restores their tolerogenic phenotype and mitigates the type 2 airway inflammation [93, 245]. In contrast, upon viral infection in the lung, the *Ifnar1* ^*−/−*^ mice show a specific reduction of pre‐cDC2‐derived CD64+ inflammatory cDC2s in a cDC‐intrinsic manner [49]. These studies highlight the role of IFN‐I in phenotypic and functional heterogeneity in cDC2s.

GM‐CSF potently induces cDC‐like cells from monocytes and CDPs in the bone marrow cell culture in vitro [26], but its concentration is much higher in the peripheral non‐lymphoid organs than in the blood and bone marrow [25]. Accordingly, mice genetically lacking GM‐CSF (*Csf2*) or its receptor subunit *Csf2rb* have a normal number of cDCs in the BM and spleen [26, 246] but they show a significant reduction of cDCs in the lung, dermis, small intestine, liver and kidney, with a more prominent reduction of cDC1s than cDC2s [25]. However, in mice exposed to dust mite allergen in the lung, blocking GM‐CSF at the sensitization with a neutralizing mAb suppresses, while supplementing recombinant GM‐CSF enhances, the ex vivo Th2‐inducing capacity of cDC2s but not cDC1s, indicating that this cytokine qualitatively alters cDC2s [46]. Interestingly, Wilkinson et al. recently reported that CD11c+ cell‐specific *Csf2rb* deletion results in a significant reduction of CD301b+ cDC2s despite an overall mild increase of cDC2s in the lungs of both naïve and dust mite‐sensitized mice, and the total lung cDC2s from these mice show reduced Treg‐inducing potential with increased Th2 potential. They also demonstrated that antigen‐specific Treg cells are specifically induced in vitro by CD301b+ cDC2s, but not CD301b− cDC2s, generated from the total bone marrow cells with FLT3L and GM‐CSF [247]. Similarly, Tadepalli et al. showed that, following conformal radiotherapy, tumor‐derived GM‐CSF drives expansion of monocytes in the tumor and their preferential differentiation into CD301b+ moDCs, which efficiently induce pTregs ex vivo and accelerate tumor growth in vivo [63]. In contrast, Ryu et al. identified a subset of CD301b+ moDCs with potent Th‐inducing capacity that arises in the spleen from monocytes upon treatment with recombinant GM‐CSF [62]. These GM‐CSF‐induced CD301b+ moDCs are required for priming OT‐II cells upon intravenous immunization with OVA protein and recombinant GM‐CSF, which sensitizes the mice for potent CD301b+ DC‐dependent type 2 lung inflammation following an intranasal rechallenge with OVA [62]. A similar subset exists in the lungs of naïve mice and also expands upon GM‐CSF treatment or after repeated exposures to fungal allergen extracts from Alternaria, but they are absent from the dermis [62], where CD301b+ cDC2s develop from pre‐cDC2s in a partially GM‐CSF‐dependent manner [61]. Thus, these studies suggest the critical role of GM‐CSF in the tolerogenicity of cDC2s, though it is currently unclear if it actively induces the tolerogenic program, or, simply regulates the number of subset‐specific precursors with developmentally imprinted tolerogenicity.

### 5.5 Regulation of CD301b+ cDC2s by Tissue‐Derived Environmental Cues

Migratory APCs capture antigens in the peripheral tissues and transport them to the dLNs in a CCR7‐dependent manner. In mouse skin, the migration of CD301b+ cDC2s precedes that of LCs and dermal cDC1s following hapten painting or intradermal immunization [58, 60, 168, 174, 248], though it remains unclear what determines their migration kinetics. Notably, however, upon exposure to papain, a cysteine protease allergen and type 2 adjuvant, the migration of CD301b+ cDC2s is triggered by sensory neuron‐derived substance P (SP) through the receptor MRGPRA1 even though CD301b+ cDC2s themselves do not seem to directly respond to papain, whereas the migration of CD103+ dermal cDC1s is not affected by SP (Figure 2B) [249]. In addition, immunization with papain induces the production of a chemokine CCL8 from the CD169+ SIGN‐R1+ interfollicular macrophages in the dLN, which directs the entry of CD301b+ cDC2s into the LN parenchyma via CCR8 signaling (Figure 2B) [171]. These studies suggest that different cDC subsets employ distinct mechanisms for engaging their migration.

Following immunization with papain, the proteolytic damage in the epithelial cells triggers the release of an alarmin IL‐33, which then stimulates group 2 innate lymphoid cells (ILC2s) to produce IL‐13. This IL‐13 promotes the migration of lung CD11b+ cDC2s to the draining mediastinal LNs and licenses CD103− CD11b+ cDC2s to produce the Th2‐attracting chemokine CCL17, which recruits memory Th2 cells to the peripheral tissue upon re‐exposure to the allergen (Figure 2C) [250]. In the skin, ILC2s constitutively produce IL‐13 and induce differentiation of CD11b+ cDC2s to CD11b−/lo cDC2s, the process dependent on KLF4 [251]. When mice are immunized in the footpad with fluorescently labeled OVA with either papain, FCA, or CpG DNA, CD301b+ cDC2s efficiently capture the antigen under all conditions, but the relative abundance of antigen‐loaded CD301b+ cDC2s in the dLN is specifically increased in the type 2 immune conditions, whereas the antigen‐loaded CD103+ cDC1s is higher under type 1 conditions [60, 169]. These results suggest that the antigen uptake and/or the migration capacity of distinct DC subsets are affected by the type of adjuvant. On the other hand, studies using pathogens have shown that CD301b+ cDC2s are the primary antigen‐transporting DC subset across distinct immune contexts, including Th1 ( *Mycobacterium smegmatis* ), Th2 (*N. brasiliensis*), and Th17 (*C. albicans*) responses [172, 192]. Thus, CD301b+ cDC2s may be equipped with multiple antigen‐uptake and pattern recognition receptors that collectively make them more efficient in handling complex antigens than other APC subsets.

As mentioned above, CD301b+ cDC2s are the primary hapten‐carrying APCs early after sensitization in the model of FITC‐induced contact hypersensitivity [58, 60, 169]. In this model, the chemical solvent di‐*n*‐butyl phthalate (DBP), not the hapten FITC, potently induces the expression of thymic stromal lymphopoietin (TSLP) from the skin and is essential for the development of hypersensitivity [252, 253]. TSLP is an epithelial‐derived cytokine that directly mobilizes human DCs and activates their Th2‐inducing potential in vitro [254]. In mice painted with FITC/DBP, TSLP selectively mobilizes dermal cDC2s to the dLN, and its receptor expression specifically in the dermal cDCs is required for the development of FITC‐specific Th2 cells and contact hypersensitivity (Figure 2B) [252, 253, 255, 256]. In contrast, hapten‐specific dermatitis induced by 2,4‐dinitrofluorobenzene (DNFB), a Th1‐dominant model of contact hypersensitivity, does not require cDC‐intrinsic TSLP signaling [255]. Intriguingly, urushiol, another Th2‐biased contact sensitizer and active component of the poison ivy allergen, also induces TSLP but not SP, whereas oxazolone, a contact sensitizer that induces a mixed Th1/Th2 response, induces SP but not TSLP [257]. Thus, these studies highlight the central role of CD301b+ cDC2s in antigen uptake and migration to the dLN across different types of immune responses.

Once CD301b+ cDC2s reach the dLN, they are preferentially positioned in the outer T cell zones near the T‐B border, a region enriched in HEVs, and are spatially segregated from CD8 T cells, migratory and LN‐resident cDC1s, and LCs, which are primarily located in the central T cell zone [58, 169, 176, 258]. LN‐resident cDC2s, by contrast, are positioned adjacent to lymphatic sinuses, where they sample antigens dispersed through the lymphatics [259, 260]. This spatial organization, also observed in the spleen and conserved in humans [261], is regulated by oxysterol sensing through the G‐protein‐coupled receptor EBI2 (Epstein–Barr virus–induced gene 2). EBI2 is predominantly expressed by both CD4+ T cells and cDC2s, guiding the positioning of CD4+ T cells [262], and, in the spleen, also cDC2s [263, 264, 265], to the outer edge of the T cell zone. While previous studies suggest that this strategic localization of CD301b+ cDC2s is important for initiating CD4+ T cell priming in the dLN, the mechanism that dictates their positioning within the LNs, including the role of CD301b+ cDC2‐intrinsic EBI2, is not fully understood [262]. However, in the lung, a recent study showed EBI2‐dependent positioning of CD301b+ cDC2s to a specific niche shaped by PDGFRα+ SCA1+ adventitial fibroblasts [266]. These studies collectively indicate the importance of tissue‐specific niche and environmental cues in regulating CD301b+ cDC2s.

## 6 Concluding Remarks

Studies discussed above corroborate functional differences among phenotypically and developmentally distinct cDC2 subsets in regulating effector Th cell differentiation. While the developmental origin determines certain aspects of their function, tissue‐derived cues also seem to have a critical impact on their phenotypic and functional maturation and diversification. In mice, CD301b+ cDC2s represent a functionally discernible subset of cDC2s shared in all peripheral organs. Studies with model antigens and adjuvants have demonstrated their specific role in timely priming of cognate CD4+ T cells and instructing Th2 cell fate while suppressing the Th1, Th17 and Tfh differentiation. However, other studies on infection and tumor models have revealed their broader roles in promoting Th17 and Treg responses, suggesting that CD301b+ cDC2s may acquire functional plasticity or further heterogeneity, either developmental or phenotypic, under a more complex setting. In addition, while functional differences between cDC subsets have been extensively investigated, their collaboration is less well studied. Understanding such complexity would be critical to fully comprehend how the compartmentalized function of cDC subsets leads to an orchestrated adaptive immune response. Lastly, recent advances in single‐cell methodologies have tremendously advanced our understanding of the heterogeneity of human cDCs and revealed conservation across species [34, 35, 37, 56, 267, 268], including the similarity between human CD301+ DCs and mouse CD301b+ cDC2s [160, 161]. While ever‐increasing new cDC subsets can be overwhelming, identifying a functionally minimum population and its commonality between mice and humans would be an important step forward toward developing clinical applications such as vaccines and immunotherapeutics targeting cDC2s and related cells.

## Conflicts of Interest

The authors declare no conflicts of interest.

## Data Availability

No data was generated in this study.
